# Important At-Sea Areas of Colonial Breeding Marine Predators on the Southern Patagonian Shelf

**DOI:** 10.1038/s41598-019-44695-1

**Published:** 2019-06-11

**Authors:** Alastair M. M. Baylis, Megan Tierney, Rachael A. Orben, Victoria Warwick-Evans, Ewan Wakefield, W. James Grecian, Phil Trathan, Ryan Reisinger, Norman Ratcliffe, John Croxall, Letizia Campioni, Paulo Catry, Sarah Crofts, P. Dee Boersma, Filippo Galimberti, José P. Granadeiro, Jonathan Handley, Sean Hayes, April Hedd, Juan F. Masello, William A. Montevecchi, Klemens Pütz, Petra Quillfeldt, Ginger A. Rebstock, Simona Sanvito, Iain J. Staniland, Paul Brickle

**Affiliations:** 1 South Atlantic Environmental Research Institute, FIQQ1ZZ Stanley, Falkland Islands; 2 Department of Biological Sciences, Macquarie University, Sydney, NSW 2109 Australia; 3 Joint Nature Conservation Committee, Peterborough, PE1 1JY UK; 4 Department of Fisheries and Wildlife, Oregon State University, Hatfield Marine Science Center, Newport Oregon, 97365 USA; 5 British Antarctic Survey NERC, High Cross, Madingley Road, Cambridge, CB3 0ET UK; 6 University of Glasgow, Institute of Biodiversity, Animal Health and Comparative Medicine, Graham Kerr Building, Glasgow, G12 8QQ UK; 7 Sea Mammal Research Unit, Scottish Oceans Institute, University of St Andrews, St Andrews, KY16 8LB UK; 8 Centre d’Etudes Biologiques de Chizé UMR 7372, CNRS-La Rochelle Université, 79170 Villiers-en-Bois, France; 9 Centre de Synthèse et d’Analyse sur la Biodiversité, Fondation pour la Recherche sur la Biodiversité (CESAB-FRB), Bâtiment Henri Poincaré, Domain du Petit Arbois, 13100 Aix-en-Provence, France; 10 BirdLife International, The David Attenborough Building, Pembroke Street, Cambridge, CB2 3QZ UK; 11 MARE - Marine and Environmental Sciences Center, ISPA—Instituto Universitário, Lisboa, Portugal; 12 Falklands Conservation, Stanley, FIQQ1ZZ Falkland Islands; 13 Center for Ecosystem Sentinels, Department of Biology, University of Washington, Seattle, WA USA; 14 Elephant Seal Research Group, FIQQ1ZZ Stanley, Falkland Islands; 15 CESAM, Departamento de Biologia Animal, Faculdade de Ciências, Universidade de Lisboa, Lisboa, Portugal; 16 DST/NRF Centre of Excellence at the FitzPatrick Institute of African Ornithology, Department of Zoology, Nelson Mandela University, South Campus, Port Elizabeth, 6031 South Africa; 17 More Energy LTD, Aberdeen, AB14 0RP UK; 18 Wildlife Research Division, Science and Technology Branch, Environment and Climate Change Canada, Mount Pearl, NL A1N 4T3 Canada; 19 Department of Animal Ecology & Systematics, Justus Liebig University, Giessen, Germany; 20 Psychology Department, Memorial University of Newfoundland, St. John’s, NL A1C 3C9 Canada; 21 Antarctic Research Trust, Stanley, FIQQ 1ZZ Falkland Islands; 22 School of Biological Science (Zoology), University of Aberdeen, Tillydrone Avenue, Aberdeen, AB24 2TZ UK

**Keywords:** Biogeography, Behavioural ecology, Conservation biology

## Abstract

The Patagonian Shelf Large Marine Ecosystem supports high levels of biodiversity and endemism and is one of the most productive marine ecosystems in the world. Despite the important role marine predators play in structuring ecosystems, areas of high diversity where multiple predators congregate remains poorly known on the Patagonian Shelf. Here, we used biotelemetry and biologging tags to track the movements of six seabird species and three pinniped species breeding at the Falkland Islands. Using Generalized Additive Models, we then modelled these animals’ use of space as functions of dynamic and static environmental indices that described their habitat. Based on these models, we mapped the predicted distribution of animals from both sampled and unsampled colonies and thereby identified areas where multiple species were likely to overlap at sea. Maximum foraging trip distance ranged from 79 to 1,325 km. However, most of the 1,891 foraging trips by 686 animals were restricted to the Patagonian Shelf and shelf slope, which highlighted a preference for these habitats. Of the seven candidate explanatory covariates used to predict distribution, distance from the colony was retained in models for all species and negatively affected the probability of occurrence. Predicted overlap among species was highest on the Patagonian Shelf around the Falkland Islands and the Burdwood Bank. The predicted area of overlap is consistent with areas that are also important habitat for marine predators migrating from distant breeding locations. Our findings provide comprehensive multi-species predictions for some of the largest marine predator populations on the Patagonian Shelf, which will contribute to future marine spatial planning initiatives. Crucially, our findings highlight that spatially explicit conservation measures are likely to benefit multiple species, while threats are likely to impact multiple species.

## Introduction

Large marine ecosystems (LMEs) are ecologically defined boundaries around the margins of continents that provide practical frameworks for ecosystem-based management at regional scales^1^. LMEs account for 75% of the global marine fish catches and contribute an estimated US$12.6 trillion in goods and services to the world’s economy^2,3^. They are, however, also hotspots of biodiversity loss and accelerated warming due to climate change^3,4^. Marine predators play important and diverse ecological roles in LMEs^5,6^. For example, seabirds and pinnipeds recycle and redistribute nutrients over large spatial scales, exert top-down control through predation and track the distribution of their prey^5,7,8^. Understanding the distribution of these taxa can therefore provide insights into processes that influence ecosystem structure and function that could otherwise be difficult to observe at regional scales. Moreover, marine predators often congregate in multi-species foraging aggregations, presumably a reflection of the spatiotemporal patchiness of their prey and perhaps the result of a higher incidence of generalism among marine predators compared to their terrestrial equivalents^9,10^. Consequently, localized threats are likely to impact multiple species, whilst spatially explicit conservation measures are likely to benefit multiple species. Hence, highlighting shared patterns in the spatial distribution of marine predators can identify ecologically significant areas that facilitates the conservation and management of not only marine predators, but also the wider ecosystem^11,12^. Advances in animal-borne tracking technology make this ever more practicable, enabling unprecedented insights into the space-use, foraging behavior and habitat preference of wide-ranging marine predators^13^. Although an explosion of movement ecology studies have facilitated the development of tracking datasets, integrated datasets from species and populations that exploit a range of habitats and ecosystem components (e.g., through diet and benthic/pelagic foraging mode) are still relatively rare, and knowledge of multi-species habitat use is typically poor, particularly in the context of LMEs^9–12,14–18^.

The Patagonian Shelf LME is one of the most productive marine ecosystems in the world^19^. Recognising its importance to biodiversity and regional economies, the Patagonian Shelf LME was the focus of a pioneering review of marine research and management^20^. The at-sea distribution of marine predators on the Patagonian Shelf has also received considerable attention and several studies have collated tracking datasets^21,22^. Nevertheless, early studies are heavily biased by the spatial usage of relatively few individuals, and until recently, limited tracking data were available for the Falkland Islands. This is a critical knowledge gap because, due to its proximity to the highly productive shelf edge and its many predator-free islands, the Falkland Islands are one of the most important locations on the Patagonian Shelf for colonial breeding marine predators. For example, the Falkland Islands are home to three quarters of the global population of black-browed albatross (*Thalassarche melanophris*), half of the global population of thin-billed prions (*Pachyptila belcheri*), half of the Atlantic population of South American fur seals (*Arctocephalus australis*), and one third of the global population of southern rockhopper penguins (*Eudyptes chrysocome*) and gentoo penguins (*Pygoscelis papua*)^23–27^. Despite the global significance of seabird and pinniped populations at the Falkland Islands, our understanding of shared patterns in their at-sea distribution is poor, recent advances notwithstanding^12^. Indeed, the movement ecology of many conspicuous species breeding at the Falkland Islands have only relatively recently been studied and the largest breeding colonies only recently tracked^28–32^. There is now an unprecedented opportunity to complement studies that have focussed on migration and individuals of unknown provenance^12,21^, and to quantify shared patterns in the at-sea distribution of colonial breeding marine predators at the Falkland Islands.

Given that many species selectively use habitats, habitat preference (selection relative to availability) can be defined by environmental variables, such as bathymetry and sea surface temperature (SST), which may provide useful proxies of resource availability^10,33^. In addition to habitat preference, colony location and density-dependent competition influence the at-sea distribution of colonial breeding marine predators^26,29,34,35^. For example, mutually exclusive colony specific foraging areas are common among colonial breeding marine predators in-part because the cost of foraging increases as a function of distance^34,36–38^. Accordingly, quantifying environmental and density-dependent factors will help to identify the drivers of spatial usage. In turn, this information can be used to predict the distribution of species more broadly, including from unsampled colonies^37,39^. In particular, Species Distribution Models (SDMs) can be used to quantify the dependence of space use on habitat (described by environmental variables) and accessibility. SDMs are increasingly being used to predict the distribution of animals around colonies that lack tracking data, using models fitted to data for observed colonies, and taking into account habitat accessibility and competition between animals from neighboring colonies^26,33,37^. Here, we aim to identify areas of high marine predator space use and diversity, which is an essential step in the management of dynamic marine ecosystems. To do so, we collated and modelled biotelemetry and biologging data for nine species of colonial breeding marine predators tracked from 21 breeding colonies at the Falkland Islands and predicted spatial usage for unsampled colonies. Given the diverse foraging guilds and foraging modes represented by these nine species, we expected that much of the area around the Falkland Islands would be used by at least some species.

## Results

### Biotelemetry and biologging data

**Figure 1:**
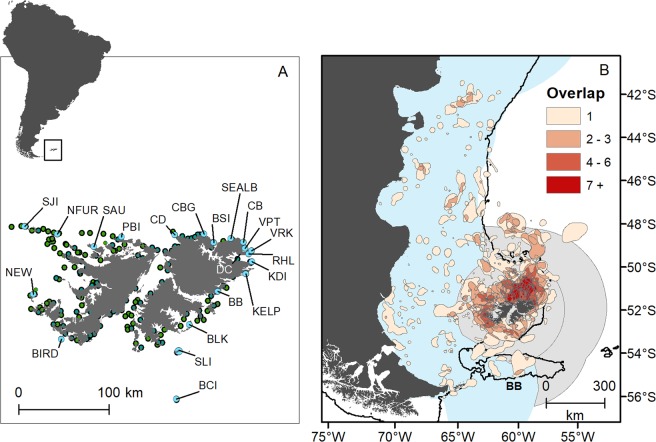
Panel (A) = locations of the 21 tracked breeding colonies (blue dots) at the Falkland Islands. Green dots are untracked colonies Panel (B) = important areas identified by overlap of 50% utilization distributions. Darker colours represent a higher number of overlapping utilization distributions. Thin black line is the 400 m bathymetric contour that marks the edge of the Patagonian Shelf, as well as the Burdwood Bank. Grey shading is the Falkland Islands Inner and Outer Conservation Zones, within which activities are managed and regulated by the Falkland Islands Government. Blue shading in the Argentinean Exclusive Economic Zone. SJI = Steeple Jason Island; NFUR = North Fur Island; SAU = Saunders Island; PBI = Pebble Island; CD = Cape Dolphin; CBG = Cape Bougainville; BSI = Big Shag Island; SEALB = Seal Bay; CB = Cow Bay; VPT = Volunteer Point; VRK = Volunteer Rocks; RHL = Rugged Hill; KDI = Kidney Island; DC = Diamond Cove; KELP = Kelp Island; BB = Berthas Beach; BLK = Bleaker Island; SLI = Sea Lion Island; BCI = Beauchene Island. BB = Burdwood Bank. Refer to Table 1 for details of the 13 groups for which utilization distributions are presented. Maps were created using R v3.5.2 and ArcMap v10.5.1.

In total, our dataset comprised locations from 1,891 foraging trips made by 686 animals (Table 1). Maximum foraging trip distance according to species ranged from 79 km for chick-rearing gentoo penguins to 1,325 km for incubating black-browed albatross (Table 1). Over 60% of sampled colonies (13 of 21) were in the north-east of the Falkland Islands (Fig. 1). The proportion of colonies from which animals were tracked for each species ranged from <10% for southern sea lions and gentoo penguins (3 of 71 and 6 of 81 colonies, respectively) to 30% for black-browed albatross (4 of 12 colonies). Rockhopper penguin and black-browed albatross foraging trips were significantly longer in distance during incubation than during chick rearing (rockhopper penguin Linear Mixed Model (LMM) F_1,75_ = 105, P < 0.001, black-browed albatross LMM F_1,65_ = 399, P < 0.001) (Table 1). Gentoo penguin foraging trips were significantly longer in distance during winter when compared to summer (LMM F_1,30_ = 19, P < 0.001).

**Table 1:** Biotelemety and biologging data collated for nine marine predators breeding at the Falkland Islands.

Species	Migratory	Season	Breeding stage	Colonies tracked [% tracked]	Individuals	Foraging trips	Trip distance (km) max [mean ± SD]
***Penguins***							
Gentoo *Pygoscelis papua*	N	Winter	Non-breeding	BB, BKI, CB, CD, PBI [6%]	25	155	479 [109 ± 80]
		Summer	Incubation/Chick rearing	NEW, CB [2%]	45	74	79 [21 ± 16]
Magellanic *Spheniscus magellanicus*	Y	Summer	Incubation/Chick rearing	BKI, CB, CD, PBI, SEALB, NEW [7%]	63	140	1,115 [298 ± 298]
King *Aptenodytes patagonicus*	N	Winter	Chick rearing	VPT	8	32	971 [295 ± 215]
Rockhopper^a^ *Eudyptes chrysocome*	Y	Summer	Incubation	CBG, PBI, SEALB, SOP [11%]	27	27	514 [216 ± 127]
		Summer	Chick rearing	BIRD, NEW, PBI, SEALB [11%]	116	185	540 [139 ± 109]
***Flying seabirds***							
Black-browed albatross^b^ *Thalassarche melanophris*	Y	Summer	Incubation	NEW, SJI, BCI, SAU [33%]	70	92	1,325 [507 ± 339]
		Summer	Chick rearing	NEW, SJI, BCI, SAU [33%]	256	699	1,235 [131 ± 147]
Sooty shearwater *Ardenna grisea*	Y	Summer	Incubation	KDI	20	43	438 [185 ± 87]
***Pinnipeds***							
South American fur seal^c^ *Arctocephalus australis*	N	Winter - female	Lactation	NFUR, VRK [20%]	9	108	674 [310 ± 216]
		Spring - female	Lactation	NFUR, VRK [20%]	9	42	940 [425 ± 295]
		Winter - male	Non-breeding	NFUR [10%]	4	34	992 [229 ± 206]
Southern sea lion *Otaria flavescens*	N	Summer - female	Lactation	CD, BS, KELP [4%]	25	93	173 [63 ± 34]
		Winter- male	Non-breeding	CD, BS [3%]	21	157	157 [88 ± 36]
Southern Elephant seal *Mirounga leonina*	Y	Summer - female	Post-breeding	SLI	10	10	497 [120 ± 42]

Data include 686 individuals and 1,891 foraging trips. ^a^Total number of rockhopper penguins tracked was 137, but some birds were tracked over both incubation and chick rearing periods. ^b^Total number of black-browed albatross tracked was 319, but some birds were tracked over both incubation and chick rearing periods. ^c^Total number of female South American fur seals tracked was 9. Please see Supplementary Information Table S1 for data sources.

**Figure 2:**
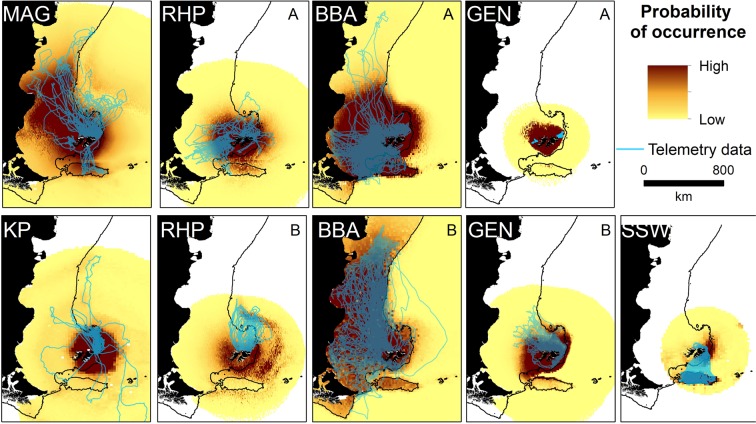
Seabird predicted habitat use (probability of occurrence, proportional to the likelihood of absences) at the Falkland Islands and available biotelemetry and biologging data (blue line). MAG = Magellanic penguin, KP = king penguin during, RHP = rockhopper penguin, BBA = black-browed albatross, GEN = gentoo penguin, SSW = sooty shearwater. A = Chick rearing, B = Incubation for rockhopper penguin and black-browed albatross, and A = Summer, B = Winter for gentoo penguins. See Supplementary Material Fig S3 for species predictions weighted by colony.

**Figure 3:**
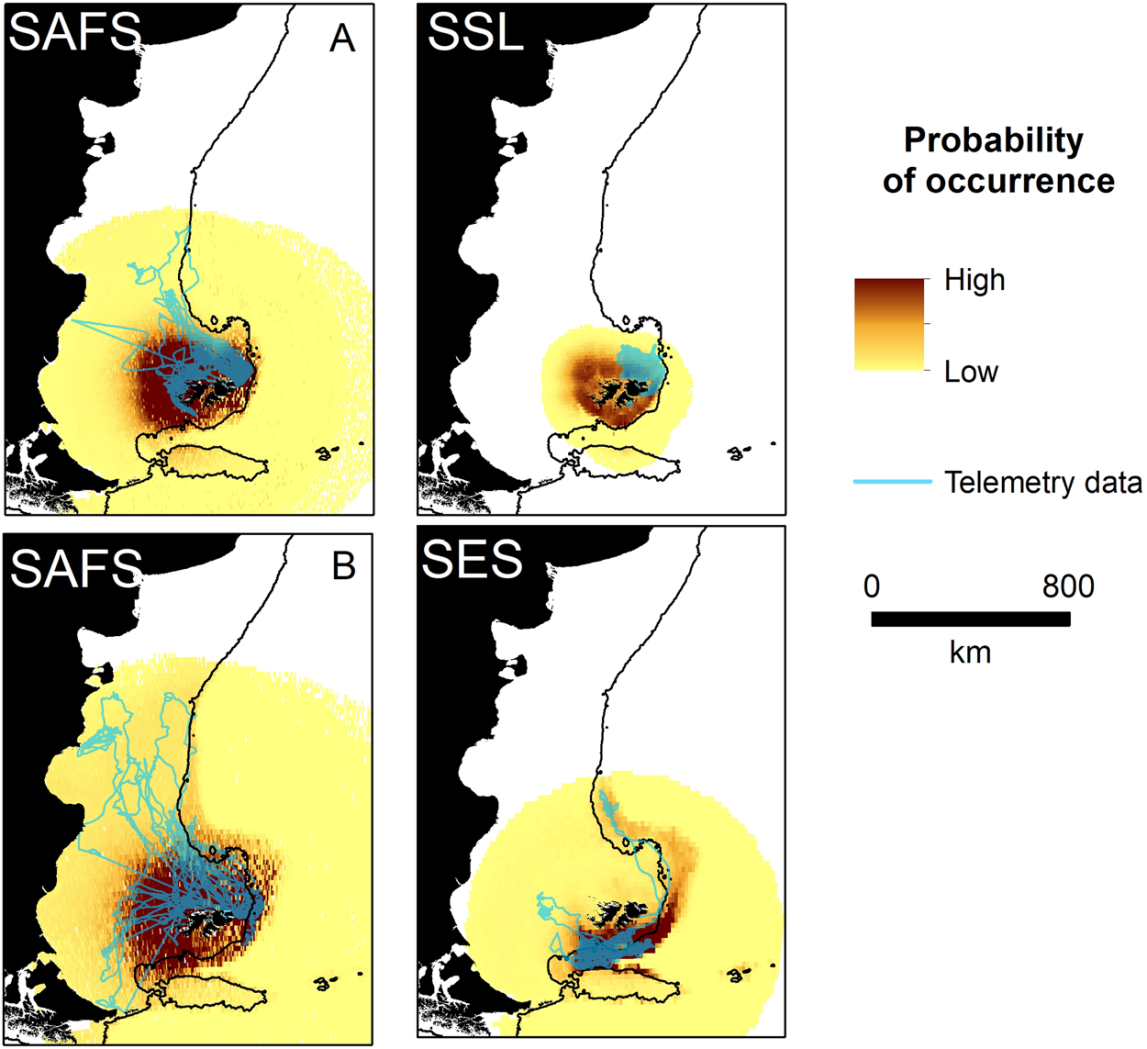
Pinniped predicted habitat use (probability of occurrence, proportional to the likelihood of absences) at the Falkland Islands and available biotelemetry and biologging data (blue line). SAFS = South American fur seal A = winter, B = Spring, SES = southern elephant seal, SSL = southern sea lion. See Supplementary Material Fig S3 for species predictions weighted by colony.

Core areas used (50% UDs), extended as far north as 42°S, 1,000 km to the north of the Falkland Islands, and as far south as 56°S, 360 km to the south. Foraging trips were, however, predominantly confined to the Patagonian Shelf and the Burdwood Bank (Fig. 1). The highest overlap of core areas, both among species and groups, was the Patagonian Shelf to the north of the Falkland Islands (Fig. 1). In total, 83% of locations were associated with bathymetric depths less than 400 m (by species, the range was 67–99% of locations), which highlighted a preference for the Patagonian Shelf and Burdwood Bank for most species. The exceptions were southern elephant seals (*Mirounga leonina*), where 68% of foraging trip locations were associated with water depths between 400 m and 800 m (i.e., shelf slope waters), and rockhopper penguins, which foraged beyond the Patagonian Shelf and slope during incubation, in water >600 m deep (Figs 2, 3). Most foraging trip locations (74%) were within the Falkland Islands Conservation Zones. The remaining locations were within the Argentinean Exclusive Economic Zone (23%), and the high seas (3%). Our representativeness analysis indicated that curves failed to saturate for some species and some colonies, implying that the empirical 50% UDs underestimated the area used. Specifically, southern elephant seals, incubating black-browed albatross, chick rearing rockhopper penguins and Magellanic penguins (*Spheniscus magellanicus*) had representative scores <75% (Table 2). Representativeness scores ranged from 60 ± 12% to 96 ± 2% (Table 2).

**Table 2:** Summary of Generalized Additive Models used for predictions, the explanatory covariates and the number of knots used in the final model. Also presented, are the area under the curve (AUC), specificity and sensitivity values associated with the final models used for predictions.

Species and breeding stage/season (group)	Explanatory covariates	AUC	Specificity	Sensitivity	Telemetry data representative value (%)
***Penguins***					
Gentoo penguin winter	Distance (k = 3) + bathymetry (k = 4) + EKE (k = 3)	0.96 ± 0.02	0.88 ± 0.08	0.95 ± 0.03	90 ± 5
Gentoo penguin summer	Distance (k = 3) + bathymetry (k = 4)	0.99 ± 0.01	0.97 ± 0.01	0.99 ± 0.01	96 ± 2
Magellanic penguin	Distance (k = 3) + bathymetry (k = 3) + SST (k = 5)	0.93 ± 0.07	0.79 ± 0.15	0.96 ± 0.03	70 ± 17
King penguin	Distance (k = 3) + SST (k = 3) + SLA (k = 3) + EKE (k = 3)	0.90	0.92	0.72	93
Rockhopper penguin incubation	Distance (k = 3) + SST (k = 3) + bathymetry (k = 3) + SLA (k = 3) + slope (k = 3) + EKE (k = 3)	0.85 ± 0.05	0.71 ± 0.01	0.93 ± 0.06	80 ± 1
Rockhopper penguin chick rearing	Distance (k = 3) + slope (k = 4)	0.96 ± 0.02	0.88 ± 0.05	0.94 ± 0.09	60 ± 12
***Flying seabirds***					
Black-browed albatross incubation	Distance (k = 3) + bathymetry (k = 4) + SST (k = 5)	0.93 ± 0.01	0.85 ± 0.01	0.97 ± 0.03	93 ± 6
Black-browed albatross chick rearing	Distance (k = 4) + bathymetry (k = 3)	0.99 ± 0.01	0.94 ± 0.02	0.96 ± 0.03	61 ± 14
Sooty shearwater	Bathymetry (k = 3) + SLA (k = 3) + distance (k = 3) + SST (k = 3)	0.94	0.85	0.92	92
***Pinnipeds***					
South American fur seal winter	Distance (k = 3) + bathymetry (k = 4) + EKE (k = 4)	0.91 ± 0.06	0.74 ± 0.17	0.95 ± 0.05	90 ± 11
South American fur seal spring	Distance (k = 4) + bathymetry (k = 4) + SST (k = 3) + SLA (k = 3)	0.86 ± 0.01	0.67 ± 0.05	0.99 ± 0.01	61 ± 5
Southern sea lion	Distance (k = 3) + bathymetry (k = 4)	0.93 ± 0.02	0.83 ± 0.05	0.97 ± 0.01	90 ± 5
Southern elephant seals	Bathymetry (k = 3) + distance (k = 5) + SST (k = 3) + SLA (k = 3) + EKE (k = 4) + slope (k = 4)	0.95	0.90	0.90	72

Finally, the representative values associated with biotelemetry and biologging data are presented (see methods for details – if multiple colonies were tracked, mean values are reported). High representative values >75 (%) indicate that biotelemetry and biologging data were considered sufficiently representative of the expected space use if more individuals were tracked. SST = Sea Surface Temperature, SLA = Sea Level Anomaly, EKE = Eddy Kinetic Energy. All values are mean ± SD.

### Habitat modelling

**Figure 4:**
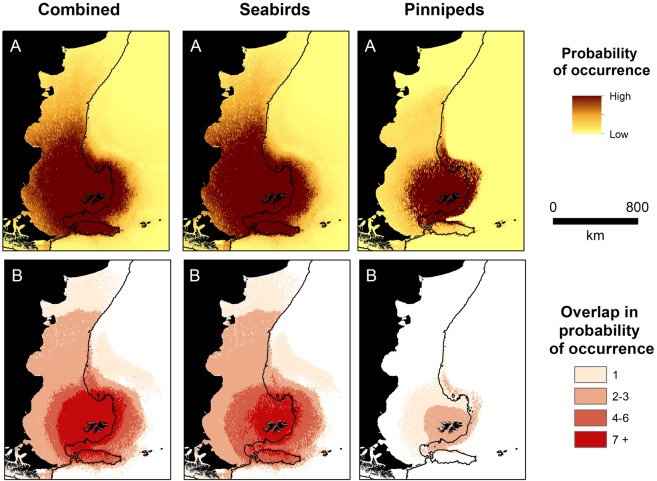
Combined species plots of (**A**) cumulative probability of occurrence (**B**) overlap in probability of occurrence between groups (see Table 2 and Figs 2 and 3 for details of predictive models) and for seabirds and pinnipeds separately.

**Figure 5:**
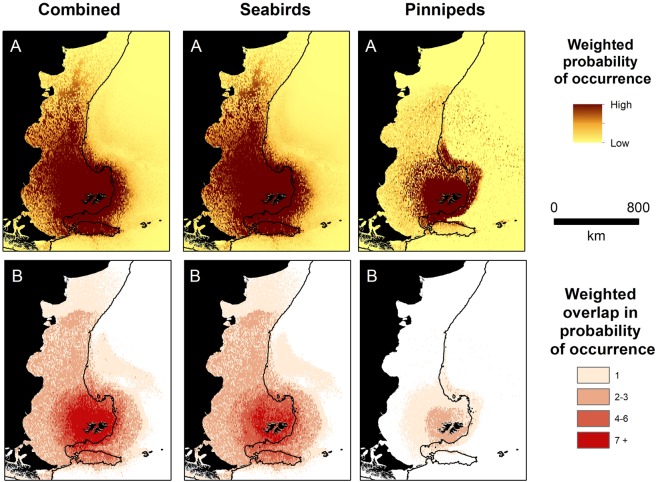
As in Fig. 4, but weighted by colony size. Plots of (**A**) cumulative probability of occurrence (**B**) overlap in probability of occurrence.

Area Under the curve (AUC) scores ranged from 0.85 to 0.99 and indicated that our models adequately described the location data (Table 2). Moreover, specificity (range 0.67–0.97) and sensitivity values (range 0.72–0.99) indicated that models predicted space use accurately (Table 2). Of the seven candidate explanatory covariates considered, distance from the colony was retained in all models, capturing a negative relationship with space use (Table 2). Bathymetry was retained in all models except those for king penguins (*Aptenodytes patagonicus*) (Table 2). Occurrence decreased with distance from the breeding colony in all species and breeding stage/season (hereafter referred to as group, see Table 2), and typically decreased with bathymetric depth (Supplementary Information Fig S1). Black-browed albatross and Magellanic penguins had the most extensive predicted distribution, while the predicted distribution of chick rearing gentoo penguins and southern sea lions were comparatively small (Figs 2, 3). The predicted distribution of all species and groups, except rockhopper penguins during incubation, were concentrated on the Patagonian Shelf or shelf slope (Figs 2, 3). Models for species that travelled extended distances typically included dynamic environmental variables, in particular sea surface temperate. In contrast, for species that predominantly foraged near-shore, such as chick-rearing gentoo penguins, dynamic environmental variables were not retained in models. When all species and groups were combined, the probability of occurrence was high across a large area of the Patagonian Shelf (>1 million km^2^), which included the coastlines of Patagonia and Argentina, as far north as the Peninsula Valdés (Fig. 4). The Patagonian Shelf around the Falkland Islands and the Burdwood Bank had the highest areas of overlap in probability of occurrence and therefore, the highest predicted diversity (Fig. 4). Similar results were obtained when weighting the predictive outputs by colony size (Fig. 5). Individual species plots weighted by colony size are presented in Supplementary Material Fig S5.

## Discussion

We analysed the most comprehensive tracking dataset compiled to date for wide-ranging marine predators breeding at the Falkland Islands. By modelling habitat selection as functions of colony distance, intra-specific competition and environmental indices, we predicted the at-sea distribution of animals from both sampled and unsampled colonies. In turn, this allowed us to identify not only high use areas for individual species but also areas likely to be used by multiple species. Our results therefore extend previous studies of marine predator distribution on the Patagonian Shelf that focus on migration or animals of unknown provenance and breeding status, and provide unprecedented insights into the distribution of colonial breeding marine predators on the Patagonian Shelf^11,12,19,40^. In particular, we provide novel insights into important areas on the Patagonian Shelf that transcend national boundaries and provide a more complete understanding of the distribution and co-occurrence of colonial breeding marine predators. For example, although the area to the east of the Burdwood Bank is highlighted as important habitat for migratory marine predators^21,41^, our predictive models highlighted the Burdwood Bank itself, which is known for its benthic biodiversity^42^, was also important habitat for marine predators breeding at the Falkland Islands. We also shed new light on the general ecology of our study species. In particular, gentoo penguin biotelemetry data revealed that individuals travelled extended distances during winter (480 km), challenging the widely held belief that gentoo penguins remain nearshore throughout their annual cycle^43^. In addition, the post-breeding movements of southern elephant seals were surprisingly short at the Falkland Islands, when compared to other breeding locations such as South Georgia (497 km versus 2,650 km, respectively) and thus they were included in our analysis of resident species^44^.

Although our predictive models generally performed well and our study improves knowledge of marine predator distribution on the Patagonian Shelf, caveats and constraints exist both with our modelling approach and the data available. Our models could have been extended by including additional terms to account for correlation structures inherent in the data, including (1) individual and colony-level slopes (random effects); (2) a term to model temporal/serial autocorrelation and (3) terms to describe spatial autocorrelation. However, including these terms has so far proved impracticable in studies that have analyzed datasets of similar size due to computational constraints and lack of convergence^10,37,45^. Even using parallel processing on multiple computers, Wakefield *et al*., (2017) found it feasible to only model colony-level random slopes. Bayesian methods such as integrated nested Laplace approximations (INLA) may soon make it possible to model spatiotemporal autocorrelation in such datasets, but at present such methods are not accessible to non-specialists. Given we used cross-validation and ROC curves, which are robust to violation of model assumptions, rather than likelihood ratio tests, we considered Generalized Additive Models (GAMs) to be a pragmatic and flexible modelling approach, suitable for our study aims that entailed predicting distributions, rather than hypothesis testing^10,37,45^. Furthermore, given our approach is relatively simple, it is accessible and easy to include more data as it becomes available, making it a potentially useful management tool when there is a need to combine ad-hoc tracking data.

In addition to model limitations, data were collected over different years, and for some species and some breeding colonies, the available tracking data were unlikely to fully represent core areas used based on our representativeness analysis. We do not know the degree to which inter-annual oceanographic variability influenced the at-sea spatial usage of our study species. Given data collection was opportunistic rather than systematic, inconsistencies in the temporal coverage of data between species could obscure potentially important shared areas of use. Ultimately, it would be ideal to track species at the same time, stratifying data collection across colonies within species, such that good geographical coverage was achieved, with more data collected from larger colonies. However, it is typically too costly to undertake simultaneous tag deployments on multiple species from multiple colonies. Finally, colony differences in habitat use are widely reported among marine predators, including those breeding at the Falkland Islands^29,35,46,47^. Spatial and temporal extrapolation are unavoidable and we cannot discount that within-species variability in foraging behaviour or habitat availability across relatively small spatial scales (tens to hundreds of km), could lead to poor model transferability^31,48,49^. Alternatively, model averages applied across a wide variety of habitats could lead to estimation bias^49^. While it would be useful for future studies to compare other modelling approaches, the spatial extent of the combined probability of occurrence, and the fact that distance was the most important variable in most models, implies that the species for which models were developed are broadly representative of the at-sea distribution of colonial breeding marine predators at the Falkland Islands.

Limitations notwithstanding, our results clearly demonstrated that the entire shelf area around the Falkland Islands, the Patagonian shelf slope and the Burdwood Bank, are important habitat for marine predators breeding at the Falkland Islands. Our findings extend that of a recent study that identified coastal waters around the Falkland Islands as important habitat for a suite of 36 seabird and pinniped species^12^. The large areas of shared spatial usage that we report, to some degree reflects the almost ubiquitous distribution of breeding colonies around the Falkland Islands for several study species, combined with long-distance movements of most study species. Furthermore, despite differences between species in foraging trip distance, dietary niches, foraging mode (e.g., benthic and pelagic), and breeding stage, most foraging trips were restricted to the Patagonian Shelf. The exceptions were southern elephant seal foraging trips, which were associated with the Patagonian shelf slope, and rockhopper penguins, which travelled beyond the Patagonian Shelf during incubation. The preference for shelf and slope waters is unsurprising given the Patagonian Shelf around the Falkland Islands is highly productive and is important habitat for marine predators migrating from distant breeding locations, such as South Georgia and New Zealand^19,41,50^. The region’s oceanography is dominated by the Falkland Current; a cold-water northward flowing current between 55°S and 37°S that originates from the Antarctic Circumpolar Current^51^. Shelf and shelf-break habitats likely provide predictable resources for marine predators breeding at the Falkland Islands, as is reported for marine predator communities in other regions such as the Kerguelen/Heard Plateau^52^. For example, the diet of albatrosses, petrels, pinnipeds, and penguins breeding at the Falkland Islands includes notothenid fish, ommastrephid squid, Falkland herring (*Sprattus fuegensis*) and lobster krill (*Munida gregaria*)^43,53–56^. The biomass of these species are typically an order of magnitude higher in neritic compared to oceanic waters, and the highest density of commercial fin-fish and squid catches also occurs on the Patagonian Shelf and slope^57–59^. Hence, the areas of overlap and high diversity that we identified are likely to represent high levels of marine predator and prey abundance and biodiversity for not only the species that were tracked, but for species such as gulls, cormorants and petrels, which were not tracked.

Given the preference for Patagonian Shelf waters around the Falkland Islands, it is unsurprising that distance and bathymetry were important explanatory covariates shared between species. The processes underlying the relationship between space use and colony distance are far from simple. For example, considerations include the energetic cost of travel to the adult, which could be greater on the return trip if animals are transporting food, the energetic cost of fasting/thermoregulating to the partner and offspring, competition (potential or realized) and social effects, such as network foraging which could be more effective close to the colony^39^. Nevertheless, while overly simplified, distance is an outcome of time and energy constraints that is common to all central place foragers, irrespective of their environment^60^. In contrast to static covariates, dynamic environmental covariates often only marginally improved model fit, or reduced model performance, as is reported for other colonial breeding marine predators during the central-place foraging period of their annual cycle^37,39^. This likely reflects the complexity of oceanography around the Falkland Islands, or ephemeral oceanographic features that aggregate species. For example, primary productivity is influenced by, among other factors, tidal movements, meso-scale fronts, quasi-stationary eddies and regions of upwelling in both summer and winter^61^. Hence, the spatial and temporal scales of dynamic environmental covariates may be too coarse to capture the environmental conditions that influence the distribution of marine predators, particularly when they are under strong central place foraging constraints^10,62^. Alternatively, understanding how dynamic environmental variables influence animal distribution may require a longer sampling period. In addition, space use and therefore habitat preference in relation to oceanography is, to some extent, decided by colony location in central place foragers, while competition can also shape the distribution of marine predators. Although we accounted for habitat availability and included an intra-specific density metric, density was not an important explanatory covariate in our models, and the influence of parapatric and sympatric competition is not always clear^39^. Nevertheless, colony location and competition with neighbouring colonies could further obscure direct links between dynamic environmental variables and the marine predators that we studied^36,39,47^.

In addition to being important habitat for marine predators, the Patagonian Shelf around the Falkland Islands also supports commercial industries including fisheries and ship-based tourism, and developing industries such as aquaculture and hydrocarbon exploration^12^. Indeed, the Falkland Islands economy is reliant on its marine environment (>60% GDP) and therefore it is essential that ecosystem-orientated approaches and marine conservation are based on robust empirical evidence and stakeholder inclusion^12,63^. In light of the loss of biodiversity within LMEs^64^, coupled with a need to address large-scale challenges with limited budgets, there has been considerable effort in recent years to generate, collate and synthesise the baseline data required for systematic and data driven ecosystem-orientated approaches at the Falkland Islands^12,63^. Our findings highlight that spatially explicit conservation measures are likely to benefit multiple species, but the suitability of measures will be scale dependent. Similarly, threats will impact multiple species. Given the importance of the Patagonian Shelf around the Falkland Islands and its complex hydrodynamics^61,65^, a more detailed understanding of finer scale oceanography and circulation within this region, could enable a better grasp of the dynamic environmental variables that influence marine predator distribution and may facilitate more precise predictions of distribution. Similarly, predictive models would benefit from longer-term research on the inherent variability of the relevant dynamic variables. Finally, our findings highlight that the distribution of marine predators on the Patagonian Shelf are not constrained by national jurisdictions. Clearly, the conservation and management of marine predators on the Patagonian Shelf would benefit from the continued development of strategic regional initiatives that integrate data and expertise from countries responsible for managing species throughout their Patagonian Shelf range^22^. Our study has extended prior knowledge of important shared areas of marine predator use for some of the largest populations of marine predators breeding on the Patagonian Shelf. Our findings will provide the baseline data required for future marine spatial planning initiatives at the Falkland Islands and therefore contribute to the management of the Patagonian Shelf LME.

## Methods

Our focus was non-migratory movements, which included the winter movements of gentoo penguins and pinnipeds. We collated seabird and pinniped biotelemetry and biologging data from published and unpublished sources (Table 1; Supplementary Information Table S1). We also deployed biotelemetry and biologging tags (Argos satellite tags and FastlocGPS tags) on penguins (gentoo, Magellanic, rockhopper) and pinnipeds (fur seals, sea lions) between 2014 and 2017 (Supplementary Information Table S1).

### Biotelemetry and biologging data analysis

We first identified individual foraging trips based on distance to land. While this was straight forward for most species, data exploration revealed large temporal gaps in gentoo penguin Argos location data, which made trip delineation difficult. Specifically, consecutive points could have represented separate foraging trips, leading to multiple trips being classed as a single foraging trip. To identify gentoo penguin foraging trips, we determined whether gentoo penguins could have returned to land between consecutive location points using a mean swim speed of 6 km/h. We were typically only able to identify multi-day foraging trips for gentoo penguins.

Location data were projected into Lambert Azimuthal Equal Area projection prior to analysis. Argos location data, Fastloc GPS and GPS data were first speed filtered (3 m/s for swimming animals, 20 m/s for flying seabirds), to remove obvious location outliers using the R package ‘Argosfilter’^46,66^. Argos locations have variable location errors, falling into six classes (3,2,1,0,A,B). In order to more accurately estimate the locations of each Argos-tracked animal along its trajectory, we fitted a continuous-time correlated random walk model, implemented through the R package ‘crawl’ (v2.1.1)^67^. Similarly, we also used ‘crawl’ to process data from Fastloc GPS tags, which provide both Argos and Fastloc GPS locations. To increase the temporal coverage of location data, we combined Fastloc GPS and Argos data, given these data are complementary^46^. Fastloc GPS locations associated with <4 satellites were removed prior to analysis^46^. We assumed Fastloc GPS locations were true locations within the model, given that error is typically <100 m^46,68^. The model estimated a best-fit track, with locations predicted hourly. GPS locations, which in the context of our study have negligible error, were linearly interpolated to hourly intervals.

To summarize the observed distribution of individuals at-sea, we split location data into groups according to species; colony; sex (if available); breeding status (breeding, not-breeding); and where appropriate, breeding stage. We used the R package adehabitatHR to estimate the utilization distribution (UD) of each group^69^. Specifically, we calculated the kernel density of locations for each individual and then normalized this to sum to one, using bathymetry as a mask to prevent UDs being estimated over land. We then averaged individual UDs by breeding colony, to create colony-level UDs. We excluded three colonies from which only one individual was tracked. To identify core areas of use, we calculated the 50% isopleth for each colony. We defined the kernel smoothing parameter *h* as the mean area-restricted search scale, which was calculated using First Passage Time analysis (assessed from 1 km to 150 km) averaged across all foraging trips for each group, and implemented using the fpt function in the R package adehabitatLT^11^. Finally, we identified shared areas of use across groups, by merging (adding) the 50% UDs of each colony, species and group^11^.

To obtain a measure of confidence in the data used for predictive modelling, we assessed whether UDs were representative of each breeding colony by calculating saturation curves based on 50% UD (i.e., core areas used). Specifically, for each species, group and breeding colony, we randomly selected an increasing number of foraging trips, with the mean area of the 50% UD and confidence interval for each step calculated from 500 iterations (a compromise between iterations and computing time). The mean area of the 50% UD was then modelled as a nonlinear asymptotic regression. We calculated a ‘representative value’ by taking the UD area of the species, and dividing it by the UD area at the asymptote^11,14^. High values (≥75%) were considered representative.

### Habitat modelling

The procedure described above produced UD estimates for animals from colonies at which tags were deployed. These UDs are unlikely to adequately reflect the space use of animals from other colonies, especially distant colonies. For example, gentoo penguins and southern sea lions (*Otaria flavescens*) breed at over 70 sites around the Falkland Islands, but were tracked from only 6 and 3 sites, respectively (Supplementary Information, Fig S2). To assess habitat use and to predict the space use of animals from unobserved colonies, we modelled habitat selection using GAMs. Although tracking data are inherently correlated within individuals, the size of our dataset made it impractical to model individual random effects, and there is arguably little to be gained by including random effects, as is reported in other recent modelling studies (please refer to Supplementary Information for additional discussion on the use of GAMs)^10,37,45^. In brief, although GAMs assume independence of errors, the cross-validation method of model selection described below, is conservative to cope with non-independence from spatial autocorrelation. By not fitting random effects for individuals, repeated trips by individuals simply become another source of non-independence that is accommodated in precautionary model selection. Hence, for the purpose of predictions and in the context of our study, GAMs were a pragmatic modelling approach.

We modelled habitat selection using logistic regression, assuming a case-control design^70^. For each observed animal location, we generated 3 at-sea pseudo-locations for each true location, which were temporally matched (date randomly selected from the associated tracking data) but randomly placed in space, within the area assumed to be accessible to animals from each colony. This we defined as being within 1.1 times larger than the maximum distance travelled by each species and group using the R package ‘rgeos’^33^. We assigned four static and three dynamic environmental covariates to each location and pseudo-location (Table 3). We selected a limited number of dynamic environmental variables that have previously been shown to influence spatial usage of at least some of the study species^33^. We used the R package ‘rerddap’ to extract sea surface temperature (SST) data (Table 3). For the remaining variables (bathymetry, slope, sea level anomaly and eddy kinetic energy) we used the R package ‘raster’ to extract values for each location and pseudo-location (Table 3). Distance was extracted from a raster of geographic distance from the colony created using the R package ‘gdistance’, which accounted for the increased cost of travel with distance away from the breeding colony, while avoiding land^71^. In order to model the potential effect of intraspecific competition, we calculated a proxy for the potential competition of animals from each colony, namely *Null density* = *population size/distance to breeding colony*^2,33^. For each colony, we then iteratively summed the density rasters from all other colonies to represent the total potential competition from animals from all colonies at each location^37^.

**Table 3:** List of static and dynamic explanatory covariates used to model and predict the distribution of marine predators breeding at the Falkland Islands.

Explanatory covariate	Source
Distance to colony (km)	Calculated using the accCost function in R package gdistance
Density of animals from other colonies	Calculated using the equation Null density = population size/distance to breeding colony^2^
Bathymetry	30 arc-second gridded bathymetric data (GEBCO_2014 Grid, GEBCO)
Slope	Calculated from GEBCO_2014 Grid in R
Sea Surface Temperature (SST)	SST, Daily optimum interpolation dataset (NOAA, 1/4°, daily)
Sea Level Anomaly (SLA)	Global ocean physics reanalysis glorys12v1 dataset (EU Copernicus, 1/12°, daily mean)
Eddy Kinetic Energy (EKE)	Calculated from the U and V zonal and meridian geostrophic currents, where EKE = ½ × (U² + V²). Global ocean physics reanalysis glorys12v1 dataset (EU Copernicus, 1/12°, daily mean)

We modelled the ratio of locations to pseudo-absences using binomial GAMs with up to 7 explanatory environmental covariates for each species, group, and colony (Table 3). Specifically, we created separate predictive models for rockhopper penguin and black-browed albatross during incubation and chick rearing stages, and separate models for gentoo penguins during winter (non-breeding) and summer (breeding), given that time and energy constraints typically differ between breeding stage and season, which in-turn may influence foraging behavior^72^ (Table 3). Differences in foraging trip distance between breeding stages were tested using LMM with individual as a random effect. We also created separate models for South American fur seals during winter and spring, given foraging behavior differs seasonally^46^. One predicative model was developed for each of the remaining species, including Magellanic penguins, which undertook both short and long foraging trips irrespective of breeding phase. Although colony information was not available for Magellanic penguins, they are distributed around much of the Falkland Islands coastline and breeding colonies often co-occur with gentoo penguins. Accordingly, we used gentoo penguin breeding colonies as a proxy for the distribution of Magellanic penguin breeding colonies. Colony predictions for southern sea lions and South American fur seals (*Arctocephalus australis*) were based on adult female data, given that the colony origin of males was unknown, and males often moved between breeding colonies (Table 3)^30,73^.

We checked for multicollinearity among the explanatory covariates using Variance Inflation Factors (VIF) and standardized covariates [(x - mean(x))/SD(x)]. To reduce the chances of overfitting, we specified cubic regression splines with shrinkage, and initially limited the number of knots to 3, increasing when fit was poor^37^. Model fit was inspected by plotting smooth terms and histograms of the raw data. We built models by forwards stepwise selection following Warwick-Evans *et al*.^37^. We assessed relative model performance by k-folds cross-validation, where k is the number of tracked breeding colonies. We also modelled distance and bathymetry as a tensor smooth to see whether this improved model fit^10^. To determine the order in which candidate covariates were added into the model, we first modelled our response variable as a function of each candidate explanatory covariate on its own, using data from *n* -1 tracked colonies. We evaluated models by predicting habitat use for the excluded breeding colony and generating a receiver operating curve (ROC) from which area under the curve (AUC), sensitivity (correctly predicted presences) and specificity (correctly predicted absences) were calculated using the R package ‘pROC’. Values of 0.5 indicated the fitted model was no better than random, whereas 1.0 indicated the model perfectly predicted space use. We added explanatory covariates to the main model in the order of AUC scores (highest to lowest). When two covariates had similar AUC values, we used each as the starting covariate, and the final model chosen was based on the model with the highest AUC values. For three species, data were only available for one breeding colony, which accounted for >90% of the population^74,75^. For these species - king penguins, southern elephant seals and sooty shearwaters (*Ardenna grisea*) - we performed cross-validation by randomly splitting datasets into training and testing datasets.

We used the best performing model for each group to predict the at-sea distribution of animals from all colonies. To estimate values that were proportional to the probability of habitat use we applied the following equation^76^:

$$\tau ({x}_{i})=\frac{\exp \{{lo}{{g}}_{e}[(1-{P}_{a})\,{P}_{u}/{P}_{a}]+\,{\beta }_{0}+{\beta }_{0}{x}_{i1+\mathrm{...}+}{\beta }_{p}{x}_{ip}\}}{1+\exp \{{lo}{{g}}_{e}[(1-{P}_{a})\,{P}_{u}/{P}_{a}]+\,{\beta }_{0}+{\beta }_{0}{x}_{i1+\mathrm{...}+}{\beta }_{p}{x}_{ip}\}}$$

Where *P*_*a*_ = the proportion of absences, *P*_*u*_ = the proportion of presences and *β*_0_… *β*_*p*_ are model coefficients. To plot the results of the GAM predictions, we first standardized the probability of occurrence for each species and group, to produce a single raster of combined (cumulative) probability of occurrence using the mosaic function in the R package ‘raster’. To calculate overlap in the probability of occurrence, for each species and group we standardized each raster cell to equal 1 where occurance was predicted, and used the mosaic function to sum raster values. Finally, we weighted species outputs by colony size to highlight important areas adjacent to the largest breeding colonies – although both weighted and unweighted results are presented. Given that the environmental covariates for pseudo-points were extracted for the same period as the tracking data, predictions are limited to the period over which tracking data were collected (see Table 1).

## Supplementary information

Supplementary Material

## Data Availability

Data are available on reasonable request made to the corresponding author, or via the South Atlantic Environmental Research Institute https://www.south-atlantic-research.org/research/data-science/data-services-metadata-catalogue/.
