# Author Correction: Important At-Sea Areas of Colonial Breeding Marine Predators on the Southern Patagonian Shelf

**DOI:** 10.1038/s41598-019-53860-5

**Published:** 2019-11-20

**Authors:** Alastair M. M. Baylis, Megan Tierney, Rachael A. Orben, Victoria Warwick-Evans, Ewan Wakefield, W. James Grecian, Phil Trathan, Ryan Reisinger, Norman Ratcliffe, John Croxall, Letizia Campioni, Paulo Catry, Sarah Crofts, P. Dee Boersma, Filippo Galimberti, José P. Granadeiro, Jonathan Handley, Sean Hayes, April Hedd, Juan F. Masello, William A. Montevecchi, Klemens Pütz, Petra Quillfeldt, Ginger A. Rebstock, Simona Sanvito, Iain J. Staniland, Paul Brickle

**Affiliations:** 1 South Atlantic Environmental Research Institute, FIQQ1ZZ Stanley, Falkland Islands; 2 Department of Biological Sciences, Macquarie University, Sydney, NSW 2109 Australia; 3 Joint Nature Conservation Committee, Peterborough, PE1 1JY UK; 4 Department of Fisheries and Wildlife, Oregon State University, Hatfield Marine Science Center, Newport, Oregon, 97365 USA; 5 British Antarctic Survey NERC, High Cross, Madingley Road, Cambridge, CB30ET UK; 6 University of Glasgow, Institute of Biodiversity, Animal Health and Comparative Medicine, Graham Kerr Building, Glasgow, G12 8QQ UK; 7 Sea Mammal Research Unit, Scottish Oceans Institute, University of St Andrews, St Andrews, KY16 8LB UK; 8 Centre d’Etudes Biologiques de Chizé UMR 7372, CNRS-La Rochelle Université, 79170 Villiers-en-Bois, France; 9 Centre de Synthèse et d’Analyse sur la Biodiversité, Fondation pour la Recherche sur la Biodiversité (CESAB-FRB), Bâtiment Henri Poincaré, Domain du Petit Arbois, 13100 Aix-en-Provence, France; 10 BirdLife International, The David Attenborough Building, Pembroke Street, Cambridge, CB2 3QZ UK; 11 MARE - Marine and Environmental Sciences Center, ISPA—Instituto Universitário, Lisboa, Portugal; 12 Falklands Conservation, Stanley, FIQQ1ZZ Falkland Islands; 13 Center for Ecosystem Sentinels, Department of Biology, University of Washington, Seattle, WA USA; 14 Elephant Seal Research Group, FIQQ1ZZ Stanley, Falkland Islands; 15 CESAM, Departamento de Biologia Animal, Faculdade de Ciências, Universidade de Lisboa, Portugal; 16 DST/NRF Centre of Excellence at the FitzPatrick Institute of African Ornithology, Department of Zoology, Nelson Mandela University, South Campus, Port Elizabeth, 6031 South Africa; 17 More Energy LTD, Aberdeen, AB14 0RP UK; 18 Wildlife Research Division, Science and Technology Branch, Environment and Climate Change Canada, Mount Pearl, NL A1N 4T3 Canada; 19 Department of Animal Ecology & Systematics, Justus Liebig University, Giessen, Germany; 20 Psychology Department, Memorial University of Newfoundland, St John’s, NL A1C 3C9 Canada; 21 Antarctic Research Trust, Stanley, FIQQ 1ZZ Falkland Islands; 22 School of Biological Science (Zoology), University of Aberdeen, Tillydrone Avenue, Aberdeen, AB24 2TZ UK

Correction to: *Scientific Reports* 10.1038/s41598-019-44695-1, published online 11 June 2019

The original version of this Article contained an error in the name of the author José P. Granadeiro, which was incorrectly given as Jose Granadeiro.

In the original version of this Article, Ginger A. Rebstock was incorrectly affiliated with ‘Falklands Conservation, Stanley, FIQQ1ZZ, Falkland Islands’. The correct affiliation is listed below.

Center for Ecosystem Sentinels, Department of Biology, University of Washington, Seattle, WA, USA.

Additionally, in the original article affiliations 15, 18 and 19 were not listed in the correct order. The correct affiliations are listed below:

Affiliation 15:

CESAM, Departamento de Biologia Animal, Faculdade de Ciências, Universidade de Lisboa, Portugal.

Affiliation 18:

Wildlife Research Division, Science and Technology Branch, Environment and Climate Change Canada, Mount Pearl, NL, A1N 4T3, Canada.

Affiliation 19:

Department of Animal Ecology & Systematics, Justus Liebig University, Giessen, Germany.

The original version of this Article also contained errors in Affiliation 20, which was incorrectly given as ‘Psycholgy Department, Memorial University of Newfoundland, St John’s, Canada’. The correct affiliation is listed below:

Psychology Department, Memorial University of Newfoundland, St John’s, NL, A1C 3C9, Canada.

Lastly, the original version of this Article contained a typographical error in the Abstract.

“Despite the important role marine predators play in structuring the ecosystems, areas of high diversity where multiple predators congregate remains poorly known on the Patagonian Shelf.”

now reads:

“Despite the important role marine predators play in structuring ecosystems, areas of high diversity where multiple predators congregate remains poorly known on the Patagonian Shelf.”

These errors have now been corrected in the PDF and HTML versions of the Article and in the accompanying Supplementary Materials file.

