# Supplementary material for: Important At-Sea Areas of Colonial Breeding Marine Predators on the Southern Patagonian Shelf
Source: Sci Rep. 2019 Jun 11;9:8517. doi: 10.1038/s41598-019-44695-1 (PMC6560117; doi:10.1038/s41598-019-44695-1)
Supplement: Supplementary file 1 — Supplementary Material [file 41598_2019_44695_MOESM1_ESM.docx]

Supplementary material

IMPORTANT AT-SEA AREAS OF COLONIAL BREEDING MARINE PREDATORS ON THE SOUTHERN PATAGONIAN SHELF

Alastair M. M. Baylis^1,2,^ Megan tierney^1,3^, Rachael A. orben^4^, Victoria Warwick-Evans^5^, Ewan Wakefield^6^, W. James Grecian^1,7^, Phil trathan^5^, Ryan Reisinger^8,9^, Norman Ratcliffe^5^, John Croxall^10^, Letizia Campioni^11^, Paulo Catry^11^, Sarah Crofts^12^, P. Dee Boersma^13^, Filippo Galimberti^14^, José P. Granadeiro ^15^, Jonathan Handley^16^, Sean Hayes^17^, April Hedd^18^, Juan F. Masello^19^, William A. Montevecchi^20^, Klemens Pütz ^21^, Petra Quillfeldt^19^, Ginger A. Rebstock^13^, Simona sanvito^14^, Iain J. staniland^5^ & Paul Brickle^1,22^

^1^South Atlantic Environmental Research Institute, FIQQ1ZZ, Stanley, Falkland Islands.

^2^Department of Biological Sciences, Macquarie University, Sydney, NSW, 2109, Australia.

^3^Joint Nature Conservation Committee, Peterborough, PE1 1JY, UK.

^4^Department of Fisheries and Wildlife, Oregon State University, Hatfield Marine Science Center, Newport, Oregon, 97365, USA.

^5^British Antarctic Survey NERC, High Cross, Madingley Road, Cambridge, CB3 0ET, UK.

^6^University of Glasgow, Institute of Biodiversity, Animal Health and Comparative Medicine, Graham Kerr Building, Glasgow, G12 8QQ, UK.

^7^Sea Mammal Research Unit, Scottish Oceans Institute, University of St Andrews, St Andrews, KY16 8LB, UK.

^8^Centre d’Etudes Biologiques de Chizé UMR 7372, CNRS-La Rochelle Université, 79170, Villiers-en-Bois, France.

^9^Centre de Synthèse et d’Analyse sur la Biodiversité, Fondation pour la Recherche sur la Biodiversité (CESAB-FRB), Bâtiment Henri Poincaré, Domain du Petit Arbois, 13100, Aix-en-Provence,

France.

^10^BirdLife International, The David Attenborough Building, Pembroke Street, Cambridge, CB2 3QZ, UK. ^11^MARE - Marine and Environmental Sciences Center, ISPA—Instituto Universitário, Lisboa, Portugal. ^12^Falklands Conservation, Stanley, FIQQ1ZZ, Falkland Islands.

^13^Center for Ecosystem Sentinels, Department of Biology, University of Washington, Seattle, WA, USA. ^14^Elephant Seal Research Group, FIQQ1ZZ, Stanley, Falkland Islands.

^15^CESAM, Departamento de Biologia Animal, Faculdade de Ciências, Universidade de Lisboa, Portugal

^16^DST/NRF Centre of Excellence at the FitzPatrick Institute of African Ornithology, Department of Zoology, Nelson Mandela University, South Campus, Port Elizabeth, 6031, South Africa.

^17^More Energy LTD, Aberdeen, AB14 0RP, UK.

^18^ Wildlife Research Division, Science and Technology Branch, Environment and Climate Change Canada, Mount Pearl, NL, A1N 4T3, Canada.

^19^Department of Animal Ecology & Systematics, Justus Liebig University, Giessen, Germany.

^20^Psychology Department, Memorial University of Newfoundland, St John’s, NL, A1C 3C9, Canada.

^21^Antarctic Research Trust, Stanley, FIQQ 1ZZ, Falkland Islands.

^22^School of Biological Science (Zoology), University of Aberdeen, Tillydrone Avenue, Aberdeen, AB24 2TZ, UK.

*corresponding author: [al_baylis@yahoo.com.au](mailto:al_baylis@yahoo.com.au)

**Methods**

*Animal handling*

We collated telemetry data from published and unpublished sources, but also deployed biologging tags on penguins and seals. Penguins were captured by hand or in a hoop net and manually restrained. Tags (Wildlife Computer SPOT 275B) were attached to the back feathers using Tesa® tape (4651) and cyanoacrylate glue (Loctite®401). Pinniped captures have been described in detail ^1,2^. Briefly, fur seals and sea lions were chemically restrained using tiletamine-zolazepam (Zoletil, Virbac; 1.5 – 3.0 mg/kg), remotely administered using darts (Pneu dart) and a CO2-powered tranquiliser gun (Dan Inject JM Standard). Anaesthesia was induced (in the case of fur seals) and maintained using isoflurane delivered via a portable gas anaesthetic machine (VOC Rota Flush, Medical Developments International). Tags (Wildlife Computers TDR10-F tags) were glued to the pelage between the shoulder blades of the animals using a two-part epoxy (Devcon® 5-minute epoxy).

*Data Analysis*

While it is advantageous to include a random intercept term, it is worth pausing to consider what the random effect is actually telling us. The null intercept in habitat preference models is actually user-defined and invariant among individuals. For example, if we follow the rule of 1 observation to 3 pseudo absences, as we have done, all individuals have an intercept of 0.25^3^. Once habitat covariates are fitted, it could be useful to fit random slopes for individuals given some animals may have different habitat preferences to others. This is biologically plausible and interesting, but fitting random slopes is difficult to implement owing to the complexity of the model (computation time, lack of convergence, mixed effects models do not predict into new datasets when random effect levels differ)^4,5^. If the model includes random slopes, then random intercepts may also be required (those with positive slopes will have lower intercepts than those with negative), although by standardizing covariates, as we have done, the intercepts are likely to be similar. However, if the model does not include random slopes for individuals, then there is little to be gained by the inclusion of random intercepts. Rather than a mean with the same values for each individual, we have a slope with the same intercept for all individuals. Indeed, it is unclear where the variation in the intercepts could arise. A main reason to fit a random effect is to enable the df to be counted more appropriately - but we use cross-validation and ROC curves rather than likelihood ratio tests. Although Generalized Additive Models assume independence of errors, the cross-validation method of model selection is conservative to cope with non-independence from spatial autocorrelation. By not fitting random effects for individuals, repeated trips by individuals simply become another source of non-independence that is accommodated in precautionary model selection. Therefore, for the purpose of predictions and in the context of our study, GAMs were a pragmatic modelling approach.

**Table S1:** Data sources and funding related to published data/co-authors.

| **Species** | **Tag type** | **Date range** | **Data source***^a^* |
| --- | --- | --- | --- |
| *Penguins* |  |  |  |
| Gentoo | Argos PTT | 2014-2015 | This study (n = 25) |
| Gentoo | GPS | 2008-2012 | ^6^, J Handley unpublished data |
| Magellanic | Argos PTT | 1999-2015 | This study (n = 53). ^7,8^ |
| King | Argos PTT | 2010 | ^9^ |
| Rockhopper | Argos PTT | 1999-2015 | This study (n = 25). ^8^ |
|  | GPS | 2008-2014 | ^6,10^ |
|  |  |  |  |
| *Flying seabirds* |  |  |  |
| Black-browed albatross | Argos PTT | 1999-2006 | ^11^ |
| Black-browed albatross | GPS | 2008-2011 | ^12–14^ |
| Sooty shearwater | GPS | 2017 | E. Wakefield unpublished data |
|  |  |  |  |
| *Pinnipeds* |  |  |  |
| South American fur seal female | Argos PTT & Fastloc GPS | 2015 | This study (n=9). ^1^ |
| South American fur seal male | Argos PTT & Fastloc GPS | 2015 | This study (n=4). ^15^ |
| Southern sea lion female | Argos PTT & Fastloc GPS | 2013-2017 | ^16^, A M M Baylis unpublished data |
| Southern sea lion male | Argos PTT & Fastloc GPS | 2014-2015 | ^17^ |
| Southern elephant seal female | Argos PTT | 2010-2011 | F Galimberti and S Sanvito unpublished data |

^a^ Funding related to published data/co-authors: JG thanks FCT/MCTES for the financial support to CESAM (UID/AMB/50017/2019), through national funds. PC thanks FCT – Portugal through the strategic project UID/MAR/04292/2013 granted to MARE (with co-funding by FEDER), and the project IF/00502/2013/CP1186/CT0003. JFM and PQ were supported by the Deutsche Forschungsgemeinschaft (DFG) in the framework of the priority programme "Antarctic Research with comparative investigations in Arctic ice areas" SPP 1158 by the grant MA 2574/6-1 and grants DFG SPP 1158, QU148/5-1.


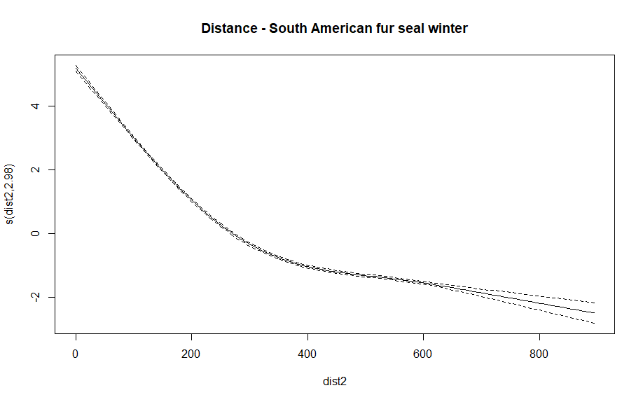

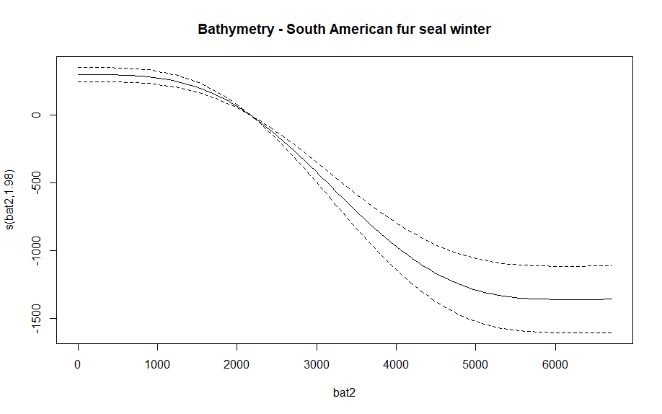

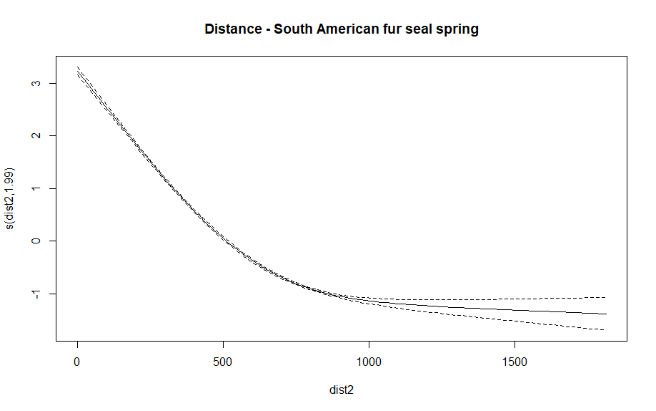

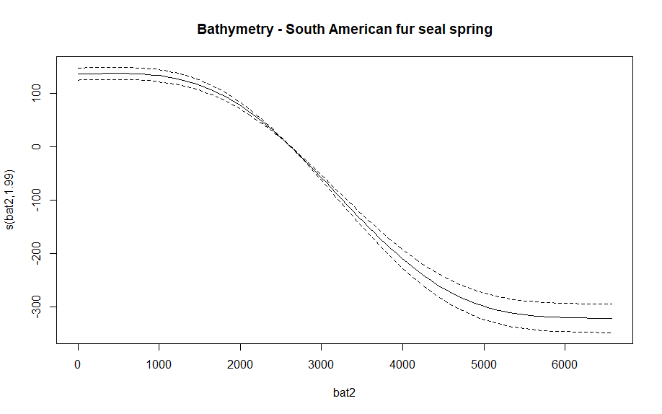

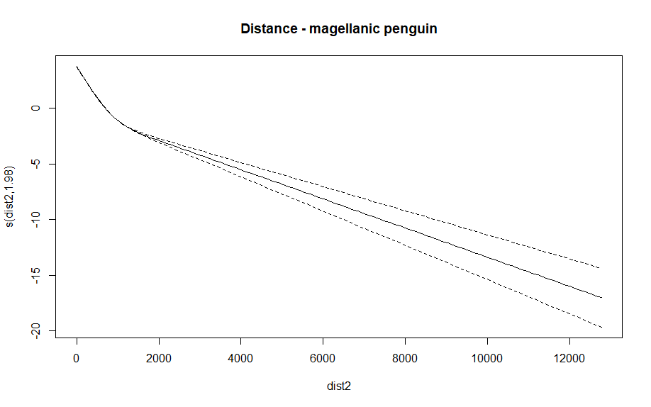

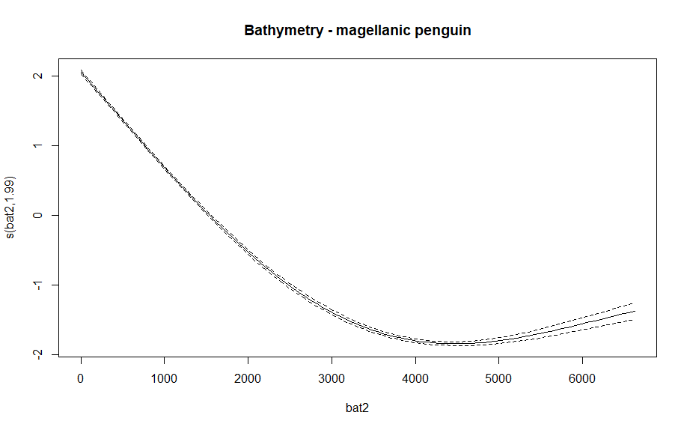

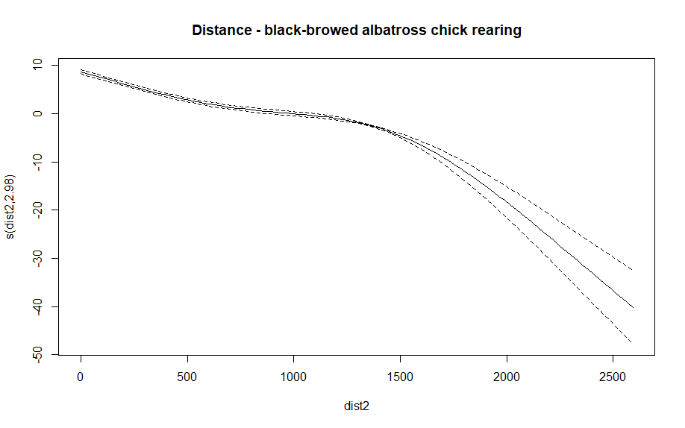

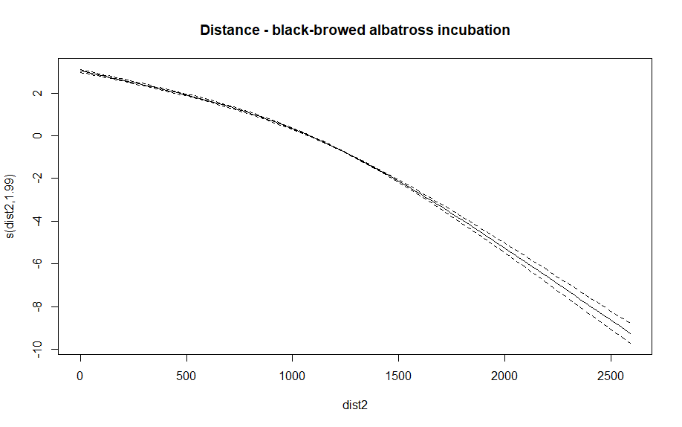

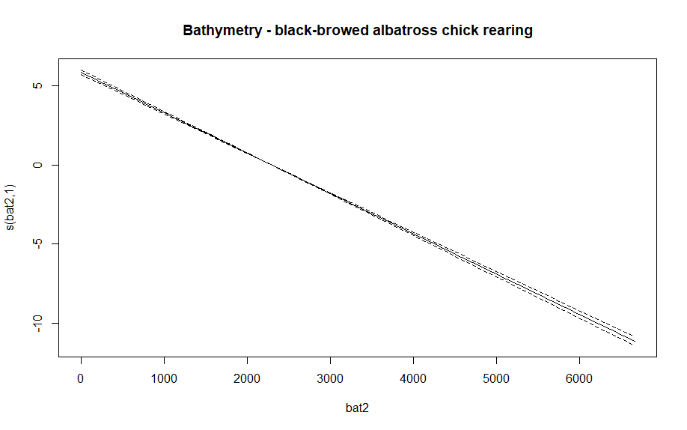

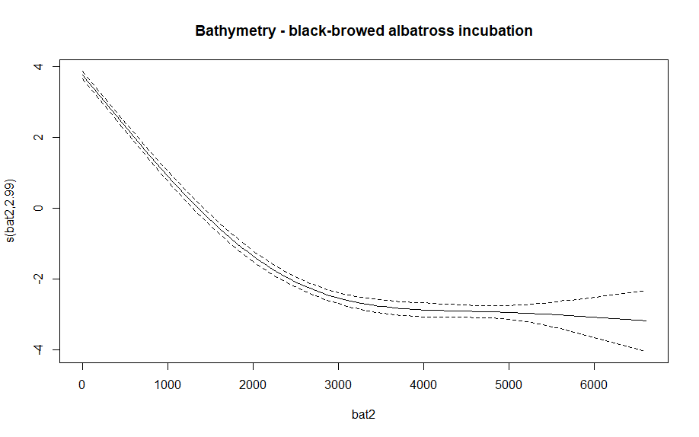

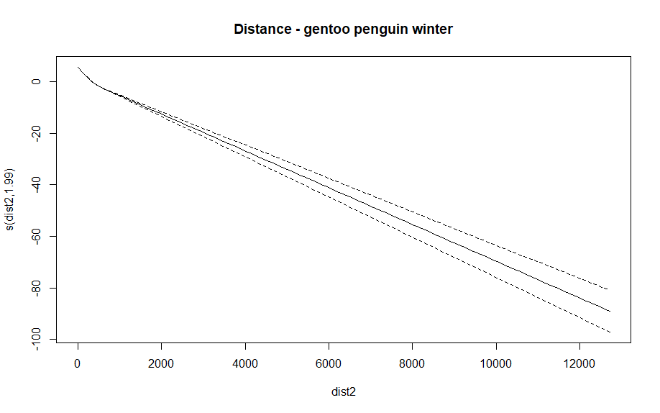

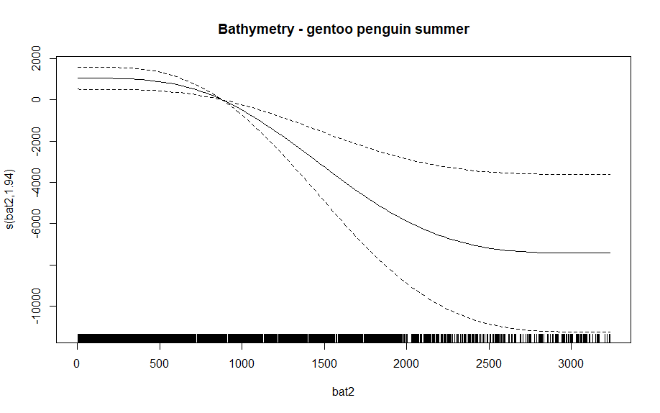

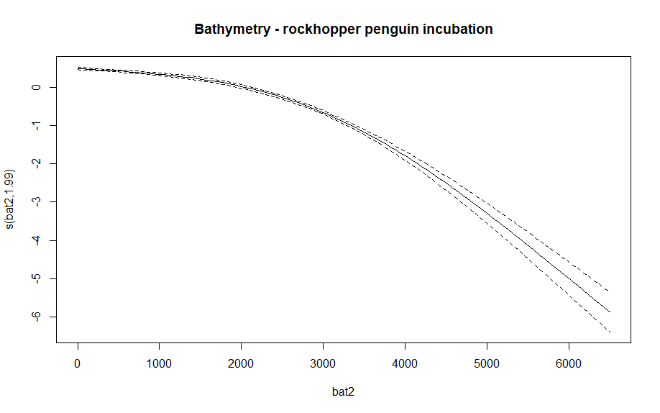

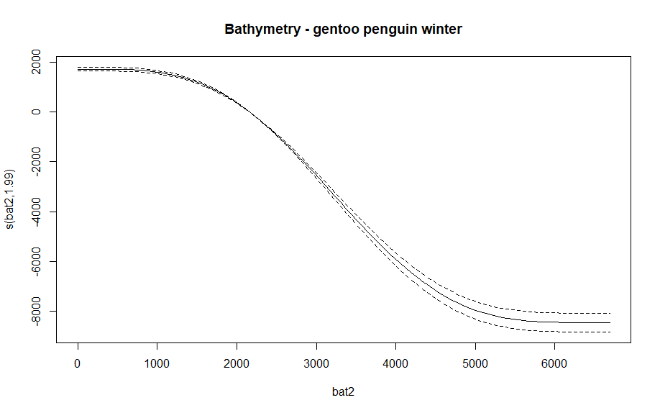

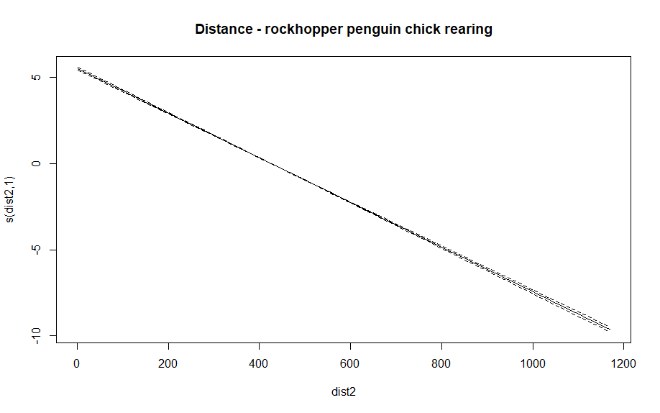

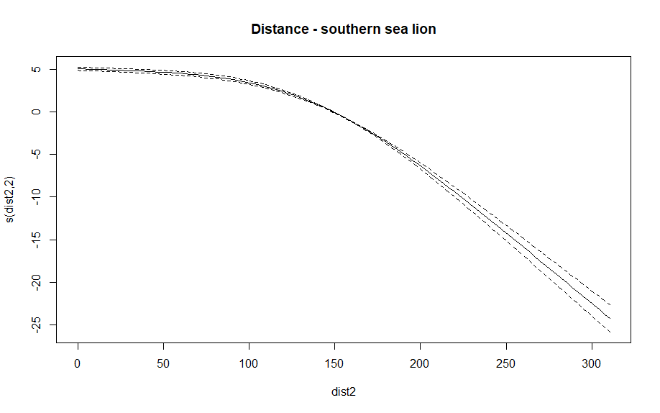

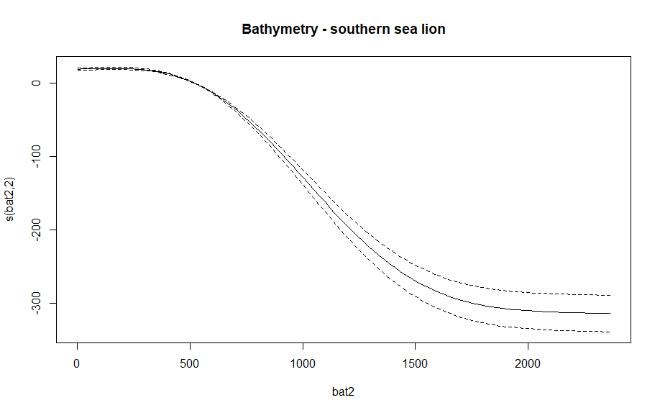

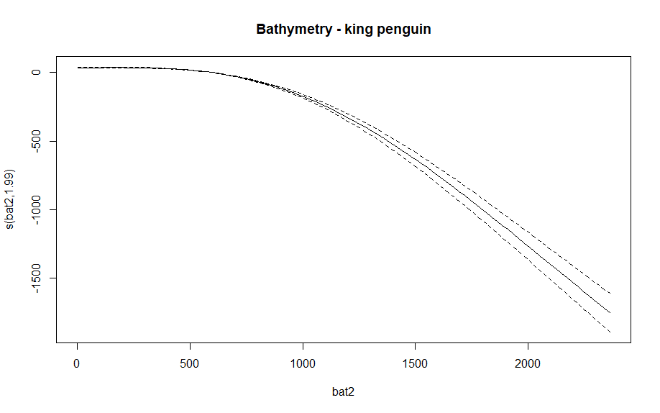

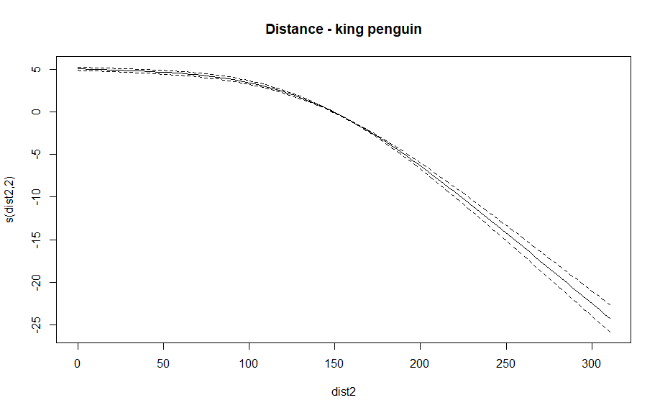

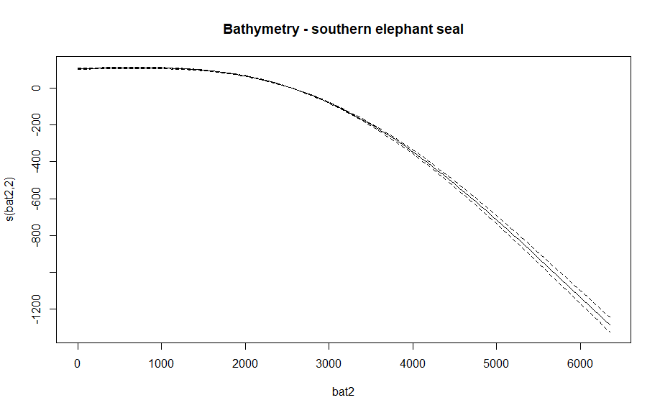

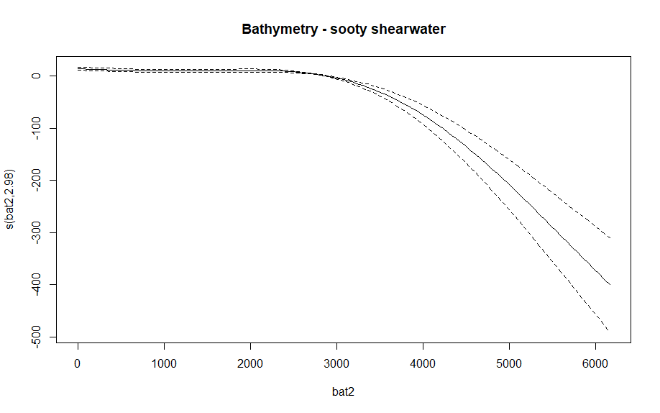

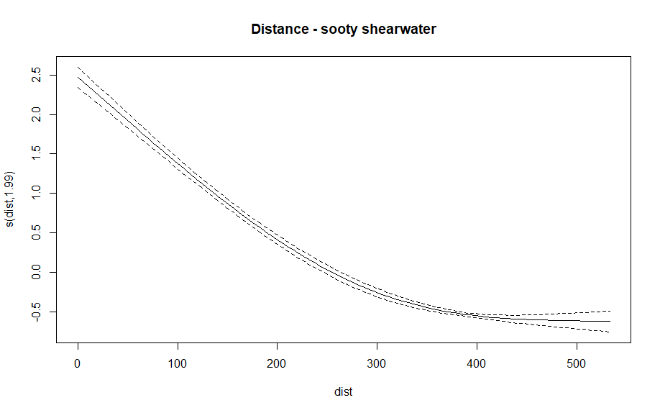

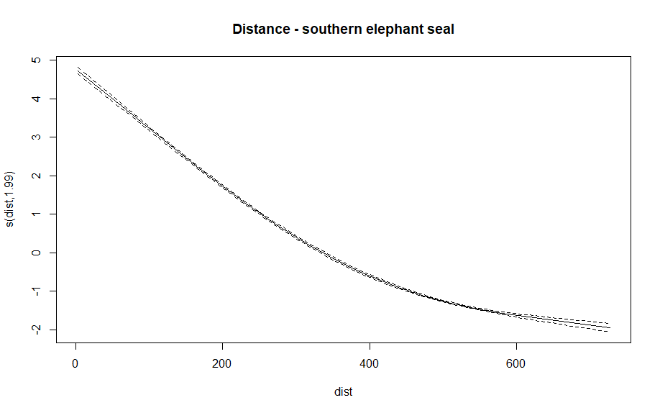

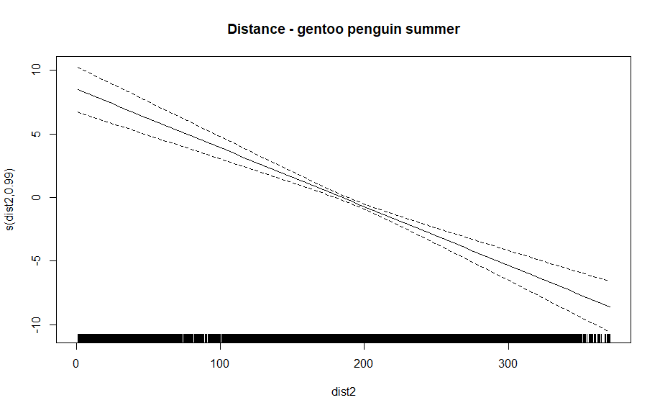


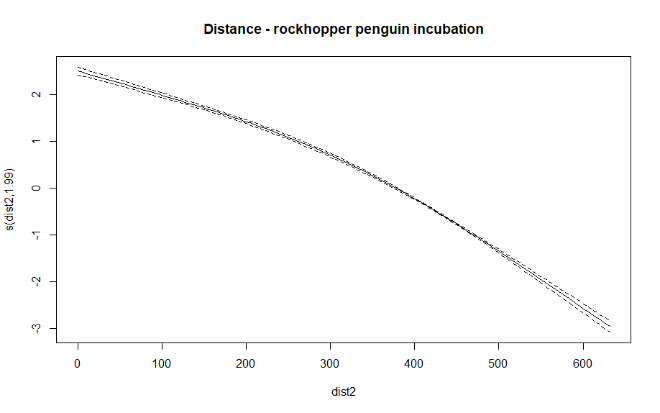


Fig S1: Generalized Additive Model smooth terms of distance and bathymetry.

^
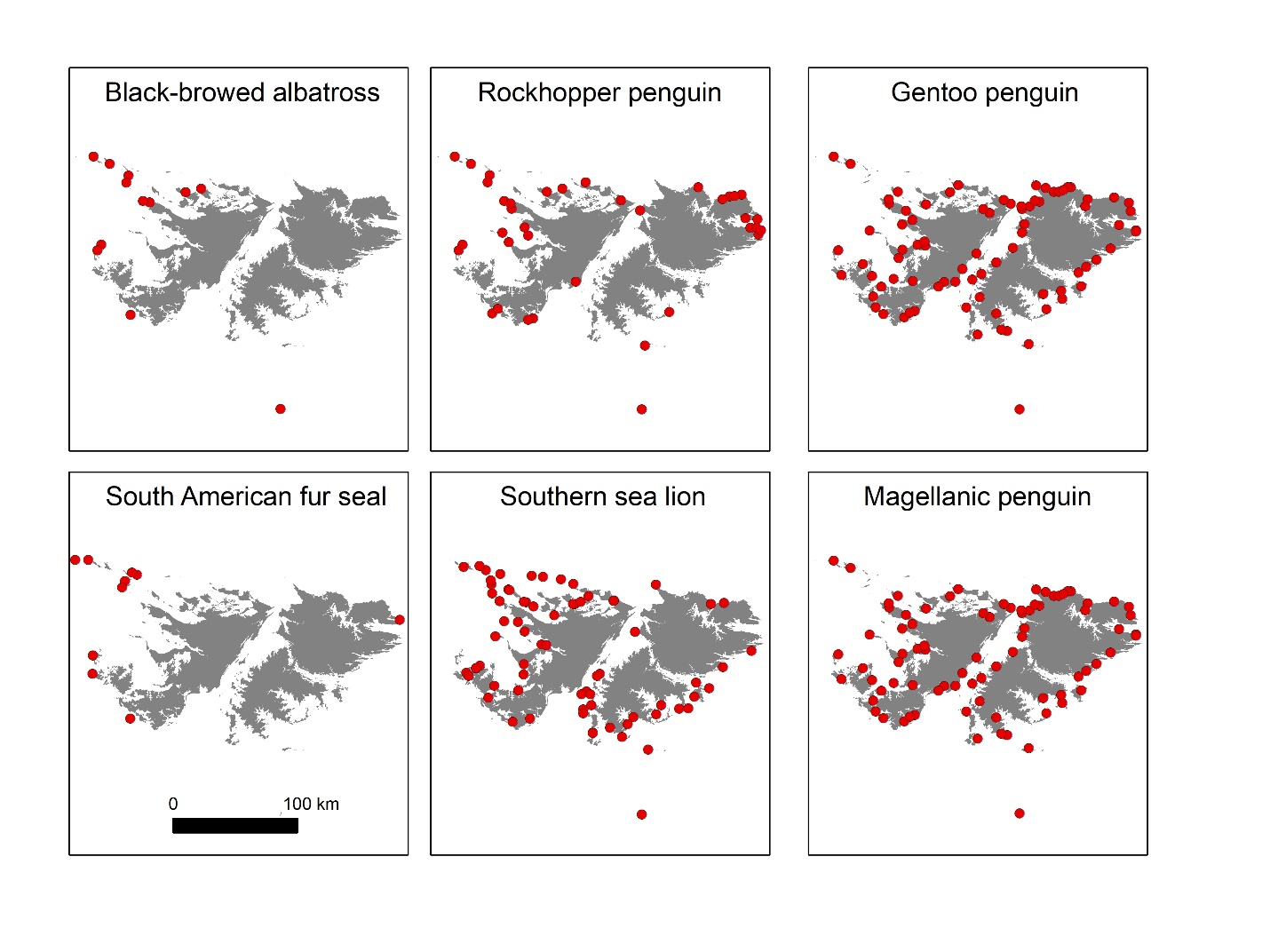
^

Fig S2: The distribution of breeding colonies for six marine higher predators breeding at the Falkland Islands (for the remaining study species, the majority of the population breeds at a single location). Note that we used the distribution of gentoo penguins to reflect the distribution of Magellanic penguins, given these species often breed in sympatry and the distribution of Magellanic penguin breeding colonies are currently poorly known.


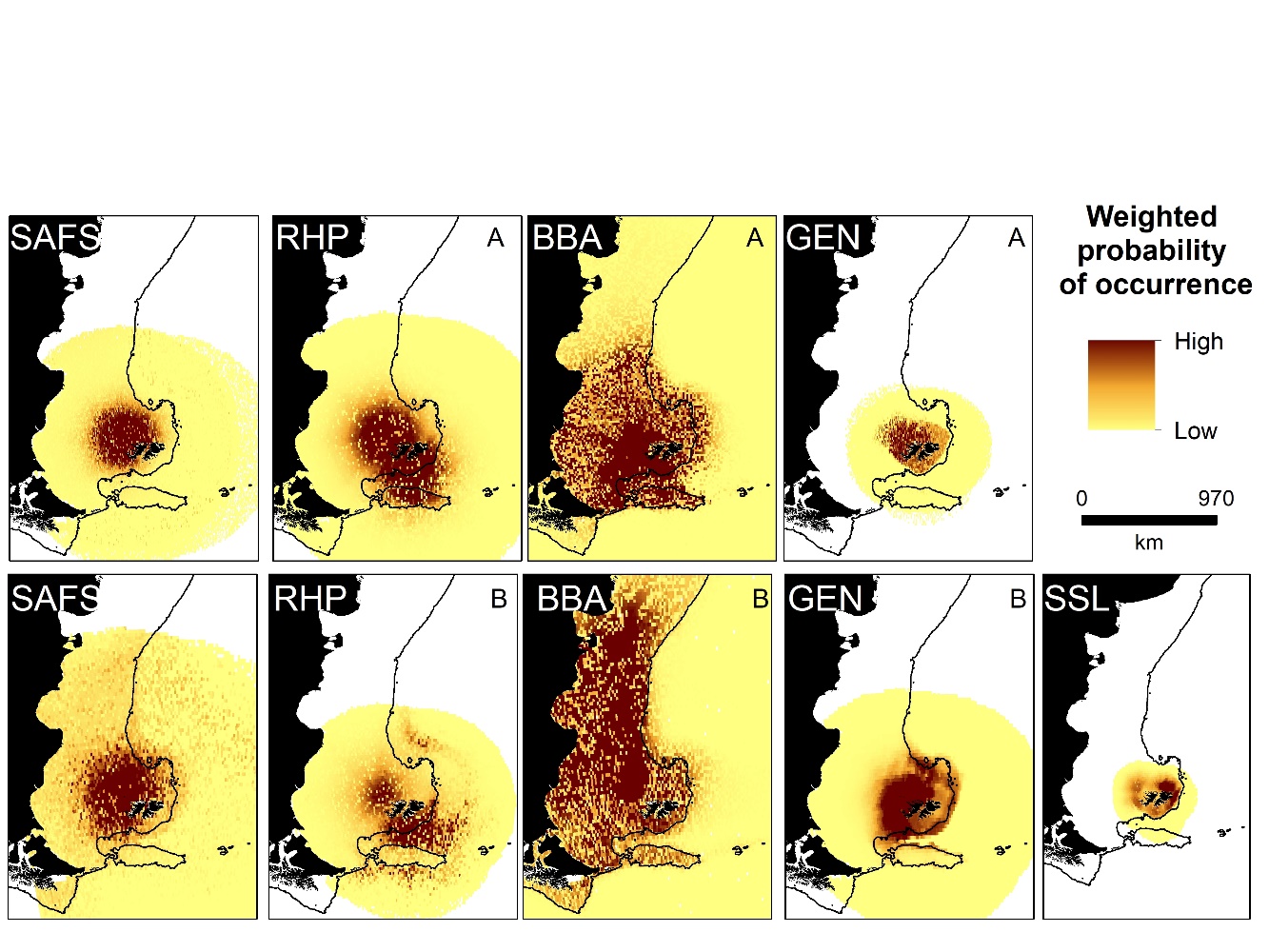


Fig S3: Predicted habitat use (probability of occurrence, proportional to the likelihood of absences) weighted by colony size. RHP = rockhopper penguin, BBA = black-browed albatross, GEN = gentoo penguin, SSL = southern sea lion, SAFS = South American fur seal. A = Chick rearing, B = Incubation for rockhopper penguin and black-browed albatross, and A = Summer, B = Winter for gentoo penguins. A = Winter, B = Spring for South American fur seals.

References

1. Baylis, A. M. M., Tierney, M., Orben, R. A., Staniland, I. J. & Brickle, P. Geographic variation in the foraging behaviour of South American fur seals. *Mar. Ecol. Prog. Ser.* **596,** 233–245 (2018).

2. Baylis, A. M. M., Page, B., Staniland, I., Arnould, J. & McKenzie, J. Taking the sting out of darting: Risks, restraint drugs and procedures for the chemical restraint of Southern Hemisphere otariids. *Mar. Mammal Sci.* **31,** 322–344 (2015).

3. Aarts, G., MacKenzie, M., McConnell, B., Fedak, M. & Matthiopoulos, J. Estimating space-use and habitat preference from wildlife telemetry data. *Ecography (Cop.).* **31,** 140–160 (2008).

4. Trathan, P. N. *et al.* Managing fishery development in sensitive ecosystems: identifying penguin habitat use to direct management in Antarctica. *Ecosphere* **9,** (2018).

5. Raymond, B. *et al.* Important marine habitat off east Antarctica revealed by two decades of multi-species predator tracking. *Ecography (Cop.).* **38,** 121–129 (2015).

6. Masello, J. F. *et al.* Diving seabirds share foraging space and time within and among species. *Ecosphere* **1,** 1–28 (2010).

7. Putz, K., Ingham, R. J. & Smith, J. G. Foraging movements of Magellanic penguins Spheniscus magellanicus during the breeding season in the Falkland Islands. *Aquat. Conserv. Mar. Freshw. Ecosyst.* **12,** 75–87 (2002).

8. Dee Boersma, P., Stokes, D. L. & Strange, I. J. Applying ecology to conservation: tracking breeding penguins at New Island South reserve, Falkland Islands. *Aquat. Conserv. Mar. Freshw. Ecosyst.* **12,** 63–74 (2002).

9. Baylis, A. M. M. *et al.* Winter foraging site fidelity of king penguins breeding at the Falkland Islands. *Mar. Biol.* **162,** 99–110 (2015).

10. Pütz, K. *et al.* Plasticity in the foraging behavior of male Southern Rockhopper Penguins (Eudyptes chrysocome) during incubation in the Falkland/Malvinas Islands. *Polar Biol.* **41,** 1801–1814 (2018).

11. Huin, N. Satellite tracking of Black-browed Albatross from Steeple Jason in the 2006 / 2007 season . Preliminary report . Nic Huin Falklands Conservation. (2007).

12. Campioni, L., Granadeiro, J. P. & Catry, P. Albatrosses prospect before choosing a home: intrinsic and extrinsic sources of variability in visit rates. *Anim. Behav.* **128,** 85–93 (2017).

13. Granadeiro, J. P., Phillips, R. a, Brickle, P. & Catry, P. Albatrosses following fishing vessels: how badly hooked are they on an easy meal? *PLoS One* **6,** e17467 (2011).

14. Catry, P. *et al.* Predicting the distribution of a threatened albatross: The importance of competition, fisheries and annual variability. *Prog. Oceanogr.* **110,** 1–10 (2013).

15. Baylis, A. M. M., Tierney, M., Staniland, I. J. & Brickle, P. Habitat use of adult male South American fur seals and a preliminary assessment of spatial overlap with trawl fisheries in the South Atlantic. *Mamm. Biol.* **93,** 76–81 (2018).

16. Baylis, A. M. M. *et al.* Diving deeper into individual foraging specializations of a large marine predator, the southern sea lion. *Oecologia* **179,** 1053–1065 (2015).

17. Baylis, A. M. M. *et al.* Habitat use and spatial fidelity of male South American sea lions during the nonbreeding period. *Ecol. Evol.* 1–11 (2017). doi:10.1002/ece3.2972
